# SNP-based high density genetic map and mapping of *btwd1* dwarfing gene in barley

**DOI:** 10.1038/srep31741

**Published:** 2016-08-17

**Authors:** Xifeng Ren, Jibin Wang, Lipan Liu, Genlou Sun, Chengdao Li, Hong Luo, Dongfa Sun

**Affiliations:** 1 College of Plant Science and Technology, Huazhong Agricultural University, Wuhan, 430070, China; 2 Biology Department, Saint Mary’s University, 923 Robie Street, Halifax, NS, B3H3C3, Canada; 3 Department of Agriculture & Food/Agricultural Research Western Australia, 3 Baron-Hay Court, South Perth, WA 6155, Australia; 4 Hubei Collaborative Innovation Center for Grain Industry, Jingzhou, 434025, Hubei, China

## Abstract

A high-density linkage map is a valuable tool for functional genomics and breeding. A newly developed sequence-based marker technology, restriction site associated DNA (RAD) sequencing, has been proven to be powerful for the rapid discovery and genotyping of genome-wide single nucleotide polymorphism (SNP) markers and for the high-density genetic map construction. The objective of this research was to construct a high-density genetic map of barley using RAD sequencing. 1894 high-quality SNP markers were developed and mapped onto all seven chromosomes together with 68 SSR markers. These 1962 markers constituted a total genetic length of 1375.8 cM and an average of 0.7 cM between adjacent loci. The number of markers within each linkage group ranged from 209 to 396. The new recessive dwarfing gene *btwd1* in Huaai 11 was mapped onto the high density linkage maps. The result showed that the *btwd1* is positioned between SNP marks 7HL_6335336 and 7_249275418 with a genetic distance of 0.9 cM and 0.7 cM on chromosome 7H, respectively. The SNP-based high-density genetic map developed and the dwarfing gene *btwd1* mapped in this study provide critical information for position cloning of the *btwd1* gene and molecular breeding of barley.

SNPs are the most abundant type of genetic markers and ideal for studying the inheritance of genomic regions^1^. Plant breeders and geneticists have benefited from the availability of tools for the rapid and cost-effective development of molecular marker-based linkage maps^2^. Linkage maps have proven to be useful for discovering, dissecting and manipulating the genes that determine simple and complex traits in crop plants^2^,^3^. Barley (*Hordeum vulgare* L.) is an important model crop for plant breeding and genetics because it is diploid and has a long history of genetics research. With the development of molecular markers, high density linkage maps of the barley have been constructed using multiple populations since the 21st century. Varshney *et al*.^4^ reported a high-density barley microsatellite consensus map with 775 SSR loci. Marcel *et al*.^5^ constructed a high-density barley linkage map using 3258 markers (RFLP, AFLP and SSR). Szucs *et al*.^6^ reported an integrated 2383-locus linkage map developed in the Oregon Wolfe Barley (OWB) mapping population based on RFLP, SSR and single nucleotide polymorphisms (SNP). SNP markers have recently become increasingly important tools for molecular genetic analysis, as single base-pair changes are the most abundant small-scale genetic variation present between related sequences of DNA^2^.

Restriction-site Associated DNA (RAD) markers detect genetic variation adjacent to restriction enzyme cleavage sites across a target genome^2^. More recent efforts have focused on adapting the RAD technique for use in NGS platforms, specifically the Illumina sequencing-by-synthesis method, to enable individual sequence based genotyping of samples^2^. The sequenced RAD marker system enjoys two favourable characteristics for high-throughput GBS. As previously mentioned, the RAD method uses restriction enzymes as a complexity reduction strategy to reduce the sequenced portion of the genome anywhere from 0.01% to 10%^1^,^2^. Furthermore, RAD protocols facilitate the creation of highly multiplexed NGS sequencing formulations. The objective of this study was to construct a RAD marker genetic map in barley for further mapping of dwarf genes.

Dwarfism is a valuable trait in crop breeding, because it increases lodging resistance and decreases damages due to wind and rain^7^,^8^. Reducing plant height has played an important role in improving crop yields. Successful use of a dwarfing gene in a breeding program is critical for developing dwarf cultivars^8^,^9^. In barley, more than 30 types of dwarf or semi-dwarf genes have been identified^8^,^9^,^10^. However, only a few dwarf genes including *uzu, sdw1* and *denso* have successfully been used in barley breeding program until now^8^. The *uzu, sdw1* and *denso* genes are located on chromosome 3HL^8^,^9^,^11^,^12^,^13^,^14^.

Huaai 11 is a new source of dwarfism, and consisted of desirable agronomic traits such as shortened stature and early maturity^8^. This dwarf phenotype was controlled by a new dwarfing gene *btwd1* mapped on chromosome 7H. The dwarfing gene *btwd1* is non-allelic with the *uzu* and *sdw1*, which have widely been used in China. The chromosome location of *btwd1* is different from those of the *uzu, sdw1/denso, br1, br2* genes and QTL *PH-7*^8^. It is a new source for broadening the genetic base of dwarfism and provides barley breeders with a new gene in China^8^. At present, the *btwd1* gene has been successfully applied in breeding program by Huazhong Agricultural University. In 2011, new barley cultivar *Huadamai 9* was registered in Hubei Province with Huaai 11 as male parent. In 2014, new barley cultivar *Huadamai 10* was registered in Anhui, Henan and Hubei Provinces with Huaai 11 as male parent. Both cultivars have high yield and good quality.

In order to efficiently use this new germplasm for barley breeding program, we have constructed a high-density genetic map based on SNP genotyping and further mapping of *btwd1* dwarfing gene using a double haploid population derived from a cross of Huadamai 6 and Huaai 11. This information could be useful for position cloning of this gene and developing new varieties for plant breeders using MAS.

## Methods

### Plant materials and field trial

A DH population including 122 lines was developed from a cross between a common feed barley cultivar Huadamai 6 and Huaai 11 using anther culture in this study. The DH population and parents were planted on the Experimental Farm of Huazhong Agricultural University, Wuhan, China. The field trials were conducted following a randomized complete block design with three replications in 2006–2008. Each of the DH and parental lines were grown in three rows in a plot of 0.6 × 1.5 m^2^. Height of plants before ripening was measured in the field from soil surface to top of the main culm (with the spike). The height was calculated as the mean of twelve plants^8^.

### Extraction of genomic DNA

The young leaves from each doubled-haploid (DH) lines and parents were collected and frozen for DNA extraction. The CTAB method was used to extract genomic DNA from about 0.6–1.0 g tissues of each accession^8^,^15^. DNA samples were electrophoresed on agarose gels for quality assurance purposes. Liquid DNA samples were normalized in water or 1 × TE to a standard concentration of 100–200 ng/μl.

### Genotyping

Frozen DNA samples were sent to the Personal Biotechnology Co., Ltd. (Shanghai, China). The libraries were quantified using Qubit fluorometer (Invitrogen), Agilent 2100 (Agilent Technologies) and real-time quantitative PCR, and then submitted for sequencing on the Illumina HiSeq2000 platform. Each marker was required to have an allele present in at least 85% of DH individuals. Marker genotypes not meeting the minimum thresholds were scored as missing data. The information of SSR markers was as previously described^16^. The segregation data for SSR markers in the same population were detected, and 68 of them were used for constructing an integrated map. The phenotyping data of dwarfing gene *btwd1* were from Ren *et al*.^8^ and listed in Table 1.

### Genetic linkage map construction

Before constructing genetic maps, SNPs were filtered by excluding those had poor quality data. Low quality SNP included those with NormR results <0.2 and SNP with large numbers of missing values (15% or more). Markers showing identical segregation patterns were also excluded, with one marker per co-segregating group retained. The DH population, consisting of 122 individuals, was utilized to construct a genetic map. The input datasets were constructed from 1,894 genotyped SNP markers and 68 SSR markers. The program Joinmap 4.0 was used to calculate the marker order and genetic distance^17^. The marker grouping process used a maximum LOD of 3.0 and 7 linkage groups were created. The Kosambi mapping function was employed for map length estimations. Markers were tested for segregation distortion using the chi-square test. Gene mapping was performed with the computer software MAPMAKER and the genetic distance (centimorgan, cM) was derived using Kosambi function^18^,^19^.

## Results

### Construction of linkage map

**Figure 1:**
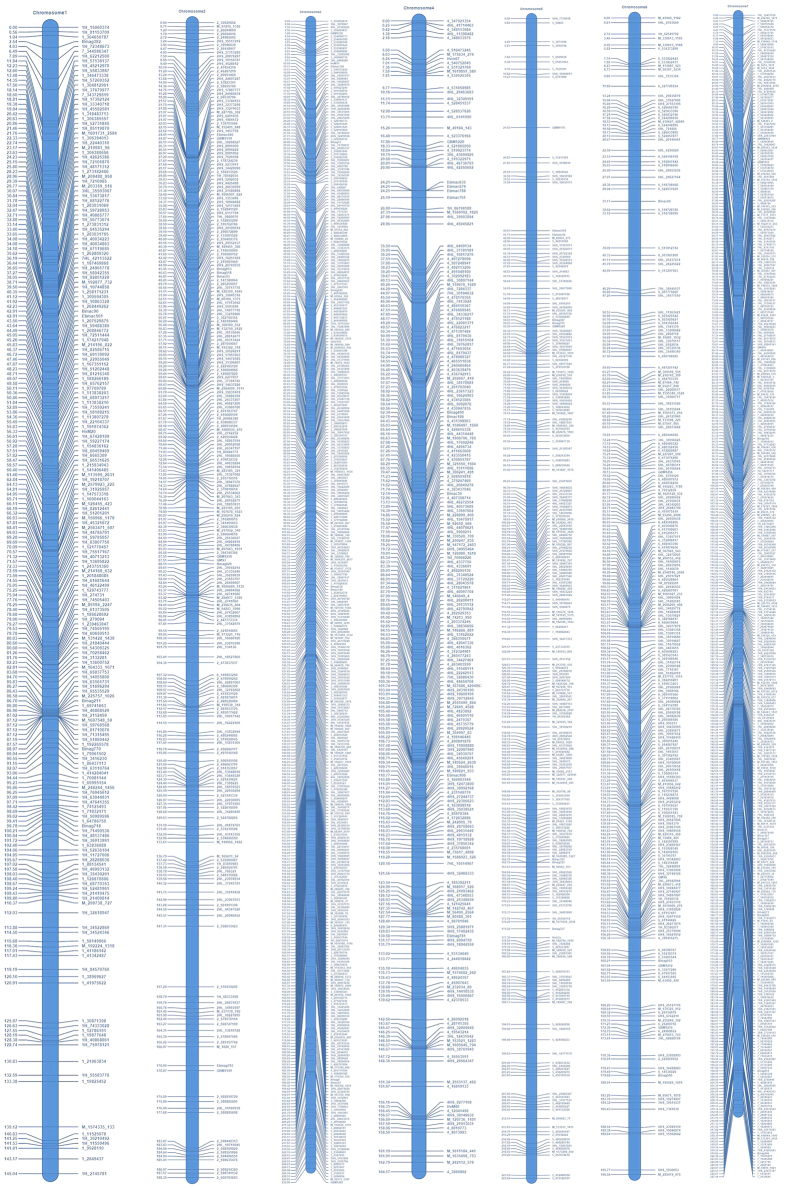
Linkage map of barley based on SNP and SSR markers derived from Huaai 11 × Huadamai 6 DH population.

38,268 polymorphic SNPs were detected between the parental lines Huadamai 6 and Huaai 11, of which 10,367 polymorphic SNPs were detected in the mapping population. All 10,367 polymorphic SNP markers showed segregation within the DH population. Low quality SNP markers excluded as a result of filtering processes comprised of 5,375 markers with inconsistent parental scores, monomorphic and more than 15% missing data. The remaining 4992 polymorphic SNP markers and 153 of the obtained before polymorphic SSR marker met the requirements for use in the construction of a genetic map. After removing some of the co-segregated markers and non-linked markers, the remaining 1894 SNP markers and 68 SSR markers were used to create 7 high-density genetic linkage groups. This high-density genetic linkage map was generated from 1,962 markers covering seven linkage groups with a total map distance of 1,375.8 cM. The averaged distance between two positions across the whole map was 0.7 cM. The number of markers on different chromosomes ranged from 209 on 1H to 396 on 7H. The genetic distance on different chromosome ranged from 145.0 cM on 1H to 230.9 cM on 3H (Fig. 1, Table 2, Table S1).

### Molecular mapping of the dwarf gene *btwd1*

**Figure 2:**
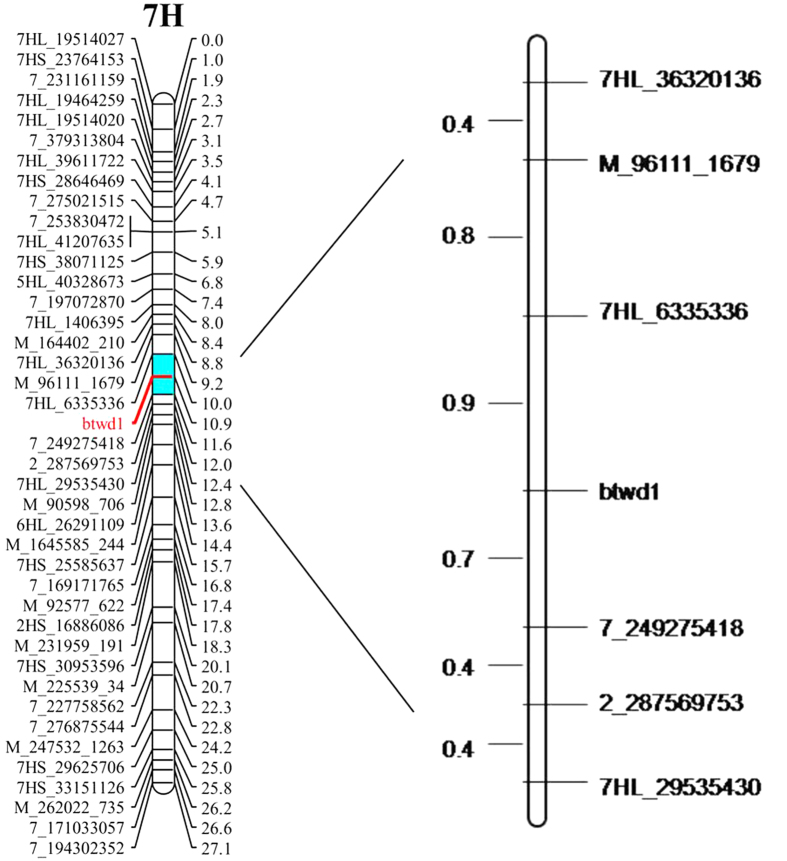
Linkage map with the dwarfing gene *btwd1* based on SNP markers on barley chromosome 7H.

The DH population was constructed from the crosses between Huaai 11 and Huadamai 6 varieties^8^. Plant height of the DH lines showed a bimodal distribution, the segregation ratio between tall and dwarf is 1:1^8^. Huaai 11 was a six-row new source of dwarf that was controlled by a recessive dwarfing gene *btwd1*, and Huadamai 6 was a two-row common feed barley cultivar^16^. Previous linkage analysis between SSR markers and plant height found that the dwarfing gene was located on the long arm of chromosome 7H, associated with the marker Bmac167 and Bmac031 at a genetic distance of 2.2 cM^8^. A set of 40 SNP markers near the SSR marker Bmac031 and Bmac167 was used to analyze the 122 individuals of DH population. The results showed that the gene *btwd1* controlling plant height in Huaai 11 was positioned between SNP marks 7HL_6335336 and 7_249275418 with genetic distance of 0.9 cM and 0.7 cM on chromosome 7HL, respectively (Fig. 2).

## Discussion

Constructing a high-density linkage map is a vital prerequisite for genetic analysis and efficient molecular breeding. For the past ten years there has been a surge in marker density and convergence toward consensus maps for barley^2^,^4^,^5^,^6^,^20^,^21^,^22^,^23^,^24^,^25^,^26^,^27^,^28^,^29^ (Table 3). In the previous published barley genetic maps, limited number of markers were included using traditional marker technologies such as RFLP, RAPD, DArT, SSR and EST based markers^4^,^5^,^20^,^21^,^30^. SNP markers have been recognized as important candidate markers due to their high abundance and relatively even distribution in barley genome. Our results demonstrated the value of RAD sequencing for the development of SNP markers and for the production of the most saturated linkage maps. RAD is one such NGS-based method which utilizes the restriction enzyme digestion of genome to reduce the genome complexity^1^. In RAD, only a small fraction of the genome is sequenced to identify many genetic markers along the genome. It is a simple and more cost-effective than other NGS methods not only for large-scale polymorphism discovery but also for genotyping-by-sequencing approach^2^. RAD sequencing data are easier to handle because less overall data are detected in comparison with whole-genome sequencing data, and therefore different individuals of populations can be easily genotyped. Using RAD sequencing approach, we identified thousands of SNPs in the Huaai 11 and Huadamai 6 DH population. In this paper, we reported the construction of a new SNP based genetic map of barley from Huaai 11 and Huadamai 6 DH population. The genetic linkage map is composed of 1894 SNP markers and 68 SSR markers. The total length of the map is 1375.8 cM with an average distance of 0.7 cM between loci (Table 2). These markers can be used for genetic diversity analysis, marker–trait association and marker-assisted selection for barley improvement.

We have used these SNP markers to map the dwarfing gene *btwd1* of barley in Huaai 11 and Huadamai 6 DH population, which previous mapped using SSR markers^8^. The results showed that the gene *btwd1* controlling plant height of Huaai 11 was positioned between SNP marks 7HL_6335336 and 7_249275418 with a genetic distance of 0.9 cM and 0.7 cM on chromosome 7HL, respectively. Our results are comparable to those in a few previous studies on dwarf and semi-dwarf genes in barley, and corresponded well with our previous result reported by Ren *et al*.^8^. The chromosome location of *btwd1* is different from the *hcm1* gene (2HL)^29^, *sdw3* gene (2HS)^31^, *uzu* gene (3HL)^8^,^12^,^29^,^32^,^33^, *sdw1/denso* geng (3HL)^8^,^14^,^29^,^34^,^35^, *br2* (4H)^8^,^36^,^37^, *ari-e* gene (5HL)^29^, *br1* gene (7HS)^8^,^36^,^37^, and also different from the plant height QTL on chromosome 1H^2^,^29^,^38^,^39^,^40^, 2H^2^,^29^,^39^,^41^,^42^, 3H^2^,^28^,^39^,^42^,^43^,^44^, 4H^38^,^39^,^40^,^43^,^45^,^46^, 5H^38^,^39^,^45^, 6H^2^,^45^, and 7H^29^,^38^,^39^,^42^,^43^,^47^, which further demonstrated that the *btwd1* is a novel gene.

Utilization of dwarfing genes in barley breeding programs has greatly increased barley yields, particularly in Asia and Europe^8^,^47^. In barley, only a few dwarf genes have been exploited in barley breeding. The dwarf germplasm Huaai 11 is a new source of dwarfs for broadening the genetic base of dwarfism. Two barley cultivars with high yield and good quality were bred with Huaai 11 as the male parent. The dwarfing gene *btwd1* in Huaai 11 has widely been used in China, and provides barley breeders with a new source for barley genetic improvement. In order to efficiently use this new germplasm Huaai 11 for barley breeding program, we will verify the identified SNP markers associated with *btwd1* in the lines developed from Huaai 11. We have also constructed a large F_2_ population including 16000 lines derived from a cross of Huadamai 6 and Huaai 11. We will fine map dwarfing gene *btwd1* using this large F_2_ population and the SNP markers. In near future, we will clone the dwarf gene *btwd1* and verify its function.

In conclusion, we have constructed a high-density barley linkage map using SNP markers derived from the DH population RAD sequencing. Our study provides a valuable genetic resource for molecular markers, map-based gene cloning, MAS and the sequence assembly of the barley reference genome. At the same time, the linked SNP markers identified in the present study can provide a useful marker-assisted selection tool to transfer the dwarfing gene *btwd1* in barley breeding.

## Additional Information

**How to cite this article**: Ren, X. *et al*. SNP-based high density genetic map and mapping of *btwd1* dwarfing gene in barley. *Sci. Rep.* **6**, 31741; doi: 10.1038/srep31741 (2016).

## Supplementary Material

Supplementary Information

## Figures and Tables

**Table 1:** Phenotyping data of *btwd1* gene.

line	btwd1	line	btwd1	line	btwd1	line	btwd1	line	btwd1
1	A	26	B	51	A	76	B	101	B
2	A	27	B	52	B	77	B	102	B
3	B	28	B	53	B	78	A	103	A
4	A	29	B	54	B	79	A	104	B
5	A	30	A	55	A	80	A	105	A
6	B	31	B	56	B	81	A	106	B
7	B	32	A	57	A	82	B	107	A
8	A	33	B	58	B	83	B	108	B
9	B	34	B	59	A	84	B	109	B
10	A	35	A	60	A	85	A	110	B
11	B	36	B	61	B	86	B	111	A
12	B	37	A	62	B	87	A	112	A
13	A	38	A	63	B	88	A	113	B
14	B	39	A	64	B	89	A	114	A
15	A	40	A	65	A	90	B	115	B
16	B	41	B	66	B	91	A	116	A
17	A	42	B	67	B	92	A	117	B
18	A	43	B	68	A	93	B	118	B
19	B	44	B	69	A	94	A	119	B
20	A	45	A	70	B	95	B	120	A
21	B	46	A	71	B	96	A	121	A
22	B	47	B	72	B	97	B	122	A
23	B	48	A	73	B	98	A		
24	A	49	A	74	B	99	B		
25	A	50	B	75	A	100	B		

A: plant height <60 cm; B: plant height ≥60 cm.

**Table 2:** Genetic distance and number of markers from the high-density map.

Chromosome	Length (cM)	SNP marker	SSR marker	Total no. of markers	Average distance (cM)
1H	145.0	202	7	209	0.69
2H	188.3	239	10	249	0.76
3H	230.9	374	11	385	0.60
4H	164.2	218	12	230	0.71
5H	223.7	235	10	245	0.91
6H	196.6	241	7	248	0.79
7H	227.1	385	11	396	0.57
Total	1375.8	1894	68	1962	0.70

**Table 3:** Recently published genetic linkage maps in barley.

Population	Size	Years	Location	Types of markers	Number of markers	Map length (cM)	References
DH	150	2004	Dundee, UK	SNP, RFLP, AFLP, SSR	1237	1211	Rostoks *et al*.^22^
DH, RIL	911	2005	Canberra, Australia	DArT, RFLP, SSR, STS	2935	1161	Wenzl *et al*.^20^
DH, RIL	683	2006	Wageningen University	RFLP, AFLP, SSR, RAPD	3258	1081	Marcel *et al*.^5^
DH	315	2006	Gatersleben, Germany	SNP, RFLP, SSR	1255	1118.3	Stein *et al*.^23^
DH, RIL	654	2006	Wageningen University	SSR	755	1067.8	Varshney *et al*.^4^
DH	90	2006	Adelaide, Australia	SSR, DArT	1000	1100.1	Hearnden *et al*.^21^
DH	139	2007	Iowa State University	SNP	1596	1017	Potokina *et al*.^24^
DH	93	2008	Okayama University	SNP, STS, SSR	2948	2136	Sato *et al*.^26^
DH	92	2008	Okayama University	SNP	1116	1187.4	Sato and Takeda.^27^
DH	93	2008	University of California	SNP, DArd, STS, RFLP, SSR	2383	1280	Szucs *et al*.^6^
DH	429	2009	University of California	SNP	2943	1099	Close *et al*.^25^
DH	93	2011	Oregon State University	SNP	436	1260	Chutimanitsakun *et al*.^2^
RIL	142	2012	Kannapolis, United States	SNP, DArT, SSR	297	832.1	Islamovic *et al*.^28^
DH	182	2013	Huangzhou, China	SSR, DArT	626	1081.2	Wang *et al*.^29^
