# Supplementary material for: SNP-based high density genetic map and mapping of btwd1 dwarfing gene in barley
Source: Sci Rep. 2016 Aug 17;6:31741. doi: 10.1038/srep31741 (PMC4987670; doi:10.1038/srep31741)
Supplement: Supplementary Information [file srep31741-s1.pdf]

Supplementary Information

"SNP-based high density genetic map and mapping of *btwd1* dwarfing gene in barley"

Xifeng Ren, Jibin Wang, Lipan Liu, Genlou Sun, Chengdao Li, Hong Luo, Dongfa Sun

Supplementary Table S1

Table S1. Markers and distances of barley genetic linkage map

Supplementary Table S1

1H		2H		3H		4H		5H		6H		7H	
marker	distance	marker	distance	marker	distance	marker	distance	marker	distance	marker	distance	marker	distance
1H_15060374	0.00	2_30929892	0.00	3_555092874	0.00	4_347921354	0.00	5HS_7374618	0.00	M_45940_1182	0.00	7HL_32457559	0.00
1H_81153709	0.56	M_97210_1356	0.71	3HL_3879907	0.96	4HL_41714463	0.25	5_54803	0.84	6HL_8783649	0.81	M_242769_3871	0.40
1_364650787	1.04	2_26694609	1.20	4HL_43169025	1.50	4_348113084	0.42	5_3871196	4.49	1H_62545792	2.74	7_599421725	0.65
Bmag382	2.54	2_26694616	1.78	3_558269837	2.16	4HL_11398482	1.38	5_3910746	5.23	M_330613_1195	3.46	7_598787646	2.69
1H_72348673	4.03	2_24985605	2.72	GBM1238	3.20	4_348033575	2.18	5_6509036	7.42	M_330613_1188	4.59	7_596056370	4.01
7_344586347	6.49	2HS_16517212	3.24	3_554090773	4.14	4_516473245	5.50	5_51657943	8.38	6_534373299	5.22	7HL_15773576	4.23
1H_62212500	7.89	2_39596039	3.95	3_554090780	4.52	M_175634_274	6.03	5_7639391	9.76	6_533822424	7.74	7_595437751	5.72
1H_57138137	11.84	2_29118917	4.47	3_533626144	5.83	Hvm67	6.24	5HS_10560611	10.82	6_533822431	7.98	7_591558041	6.37
1H_45212970	12.23	2HS_37313285	4.95	3_551891621	6.08	4_540752045	7.01	5_6313908	11.27	M_174987_523	8.23	7_591226833	6.92
1H_55833987	12.60	2HS_20878297	7.50	3_551929257	6.34	4_531321769	7.36	GBM1176	21.03	M_56387_3234	8.73	M_799949_1714	7.35
1_346473338	13.48	2HS_10154317	8.56	3_550668826	6.63	M_1619851_580	7.59	5_13217441	26.83	6HL_1535364	9.86	7_588435098	9.00
1H_57269352	14.35	2HS_4528612	9.49	3HL_33685908	6.91	4_539125355	7.81	5HS_4708010	27.85	6_527745554	11.60	7_579835513	10.47
1_304812961	17.50	2_47454319	10.19	3_549980712	8.62	4_531058985	9.77	5_18844526	29.59	6HL_29835879	13.24	7HL_41355425	10.70
1H_37679977	18.14	2_46287210	11.20	3HL_8468228	9.13	4HL_29463683	10.16	5HS_10630182	30.56	6HL_37627090	15.04	7_584904294	11.59
7_343729819	18.60	2_80951468	13.24	HvM70	10.10	4HL_32300191	11.11	5_19830139	31.03	6HS_27553105	15.40	7_584878350	11.99
1H_17392124	18.81	2HS_24897287	13.68	Ebmac708	11.17	4_528451537	11.74	5HS_10630175	31.59	6_526666780	16.20	7_583744292	14.21
1H_33340718	19.38	2_53823341	14.04	3_546535222	11.53	4_528537626	12.88	Ebmac518	40.93	6_525833390	16.59	7HL_30780105	14.93
1H_45582581	19.84	2_58655700	14.36	3_544159655	12.08	4HL_6141990	13.71	Ebmatc40	41.71	6_525832746	17.21	M_5654_1031	16.00
1_318483713	20.42	2HS_17887777	14.76	3HL_21691177	12.91	M_49184_143	15.26	M_86861_975	42.46	6_524296656	17.92	M_5654_1024	16.82
1_306385557	20.63	2HS_24984434	15.28	3HL_6924929	13.97	4_523376166	16.40	5_46911009	42.96	M_210627_4294	18.53	7HL_25087539	17.46
1H_52731845	20.86	2_69538796	18.90	3HL_14026067	14.57	GBM1220	17.86	5HS_17167071	43.74	6_524246995	19.13	7_579149290	18.12
1H_85119870	21.46	2HS_33784153	21.57	3HL_32425970	15.98	4_521965050	18.33	5HS_25526871	44.45	6HL_721866	19.58	7HL_24591633	19.07

M_1601731_2584	21.76	2HS_32373258	21.75	3_541623241	17.58	4_519923774	18.64	5HS_21722342	45.34	6_526633800	19.73	7HL_24626778	19.94
1_306394013	22.33	2HS_33274533	21.88	3_541194934	17.67	3HL_43694925	19.75	5HS_2329569	46.13	6_524857877	20.13	7HL_16833964	20.69
1H_22440310	22.74	M_227786_302	21.97	3_541192201	17.76	4_516322971	20.68	5HS_15522513	46.96	6HL_20140441	20.67	M_2558454_2	21.06
M_219981_96	23.17	2HS_6201430	21.97	3HL_42780152	18.89	4HL_46730793	20.81	5HS_20364868	47.18	6HL_6256820	22.50	7HL_2460896	21.46
1_306380656	23.63	2HS_1984172	21.97	3_534961137	19.32	4HL_42250692	20.90	5_92146019	47.81	6_524146158	23.53	7HL_27281835	25.33
1H_42625386	24.23	2_139701566	22.03	3_535856638	19.60	Ebmac635	24.21	5HS_214983	48.83	6_518847442	24.45	7_575487388	25.64
1H_72101870	25.15	M_155401_848	22.15	3HL_10565488	21.14	Ebmac679	24.21	5_108529915	49.70	6_518846666	25.03	7_567206277	27.48
1H_48171312	25.80	2HS_1457758	22.62	7HS_22292244	21.97	Ebmac788	24.21	5HL_51382606	50.64	6HL_29653636	25.90	7_570371047	28.13
1_273182460	28.23	Ebmac640	24.41	3HL_38531260	22.55	Ebmac701	25.19	5HL_39677860	52.05	6HL_29627164	27.07	7_568143905	29.30
M_208488_950	28.96	GBM1119	24.87	3HL_41243504	23.55	1H_86798188	26.90	5_215461168	53.18	6_518798568	28.36	7_565646661	30.44
1H_7210965	29.39	2HL_39208527	26.11	3HL_24835298	23.92	M_1569102_1825	27.31	5_28519477	54.00	6_522937629	29.04	7_564913828	30.82
M_203359_516	29.77	2HS_8991009	26.31	3_528786733	24.28	4HL_39593094	27.96	5HL_6916210	54.99	Bmac40	31.17	7_565612902	32.36
3HL_35503007	30.07	2HS_8990224	26.51	3_528470541	25.88	4HL_45045821	28.96	5HS_24302205	55.89	6_518728726	32.62	7_565608892	32.90
1H_13673817	30.84	2HS_8991002	26.97	3_528413521	26.31	4HL_6409134	35.50	M_235501_969	56.73	6_518708998	33.19	7_563189691	34.22
1H_68122776	31.78	2HS_7525108	27.20	3_529115904	26.68	4HL_37781181	35.95	5HL_40195336	57.31	6_513912312	39.09	M_128403_0	34.62
1_283031069	32.04	2_178724239	27.61	3HL_38740410	27.59	4HL_10917270	36.24	5_133009849	58.01	6_507987808	40.33	7HL_11207206	35.05
1H_59728953	32.47	2HS_5233911	27.68	2HS_32409186	27.73	4_497278098	37.51	Bmag387	59.03	6HL_25237074	41.11	7_563346281	35.36
1H_40865777	32.71	2HS_34429090	29.17	3_525094736	27.83	4_507248941	37.88	Bmag751	59.03	6HL_28225622	41.79	7_563192425	35.54
1H_56773674	32.88	2_184213520	29.68	Bmag13	28.48	4_492113206	38.23	GBM1229	59.36	6_513207563	42.95	7_556060018	37.09
1_273831312	33.14	1H_78592035	29.85	3HL_11798617	29.05	4_491049100	39.20	5HS_16566687	60.34	7HL_38945537	46.29	5HS_22933940	37.68
1H_64535294	33.40	2HS_5292032	30.03	3HL_29995337	29.52	4_302992183	39.67	5HL_43554882	61.55	6_507775660	46.88	AWBMS22	38.77
1_283031705	33.65	2HS_4425488	30.23	3_510997641	30.17	4HL_30807144	40.13	5HS_19323912	62.71	6HL_31677510	47.20	7_549668049	40.40
1H_40034223	34.08	2HS_28630950	30.79	3_511738805	30.32	M_159676_1589	40.26	5HS_17673413	63.31	6HL_11563421	50.53	7HL_34924967	40.74
1H_40034863	34.08	2HS_21580884	31.38	3_510995799	30.45	4HL_7284337	44.29	5_351609227	64.06	6_503425542	50.98	M_205503_1634	44.67
1H_67119685	34.18	M_1606997_622	32.42	3HL_15958297	30.94	7HL_30194632	44.97	5HS_10311601	64.44	6_503925621	51.76	7_540653807	45.10

1_262809320	35.62	2HS_1353446	33.52	3HL_13955756	31.10	4_478170355	45.54	5HL_47892947	64.60	6_504356044	52.09	7_542100185	46.00
7HL_42113522	36.19	2HS_10844482	34.37	3HL_6983780	31.10	4HL_7413924	46.06	M_153633_1451	64.68	6HL_7541370	53.02	7_540657058	46.69
1_187469868	36.44	2HS_12173461	35.08	2HL_33741786	31.27	4_484110367	46.63	5HL_45327316	65.12	6HL_13208482	53.93	7_542104217	47.10
1H_24961778	36.65	2_152841129	35.83	3HL_15958290	31.63	4_476069545	47.51	5_84104995	65.88	6HL_2868718	54.05	GBM1102	49.24
1H_58042355	37.27	2HS_6117794	36.57	3HL_32354397	32.37	4HL_34336217	47.80	5HL_49177504	66.77	M_10881_1032	54.68	7_516026682	50.66
1H_82851228	38.05	7HL_9860070	37.64	3_504148850	32.64	4_478121188	48.17	5_251934708	67.24	6HL_33075117	55.32	7_536158078	51.58
M_192877_732	38.71	2_152855299	38.08	3_511749149	32.72	4HL_22081371	49.28	M_115973_1160	68.22	6HL_8182034	55.99	7_539340050	52.68
1H_10744858	39.50	2_105762766	38.59	3_504106156	35.91	4_476823217	49.57	M_236918_510	69.40	6HL_39725136	56.24	M_216504_505	52.92
1_258171231	40.35	2HS_29109012	38.66	3HL_628887	36.39	4_471397484	50.34	5HL_11830955	70.06	6HL_35490340	56.72	7HL_6657809	53.15
1_300594305	41.12	2_296072899	39.10	3HL_44939929	36.95	4HL_8179430	50.99	5HS_214990	70.58	6_495768585	57.50	M_159744_1945	55.14
1H_10863328	41.92	2_133001522	39.49	3HL_43480963	37.50	4HL_31815104	51.30	5HS_2044925	71.19	6_497251142	59.40	7_598211495	55.38
1_268449262	42.23	2_230402376	40.00	5HL_48131831	37.89	4HL_39762817	51.42	5HS_12395032	71.50	M_305199_555	61.27	7_529554189	55.53
Bmac90	42.91	2HS_29552137	40.16	3HL_22406140	38.13	4_471693054	51.53	5_66389622	71.86	M_216745_309	61.79	7_552887354	55.95
Ebmac501	42.91	M_189451_366	40.30	3HL_11772588	38.49	4HL_8179437	51.74	5HS_13258294	72.46	6_484793339	62.05	1_4088556	56.59
1_207529875	43.50	2_110610069	40.87	6HL_29119759	38.61	4_474989327	52.24	5_134943899	73.18	M_87464_742	62.21	7_523855164	57.49
1H_59488380	43.64	2_109580152	41.65	3_499436820	41.41	4_465511838	54.51	5HS_15616455	73.80	M_65217_848	62.62	7_524132075	57.85
1_208846772	44.49	2HS_16257459	42.42	3HL_7173767	41.90	4_240485464	54.90	5HS_14996024	74.29	6HL_22068577	63.31	7HL_29591288	57.96
1H_72511444	45.05	2_215081469	42.87	3HL_12267275	44.10	4_453639479	55.40	5_168012062	75.20	M_1595340_1528	63.85	1_4089724	58.01
1_174217948	45.26	2HS_26743035	44.09	3HL_12268950	44.10	4_436742913	56.22	5_147872945	75.77	6HL_39566717	64.39	M_141415_212	58.05
M_214196_822	45.83	Bmag813	45.45	3HL_40362485	45.10	M_292667_419	56.91	M_165866_1487	76.15	6HL_36831590	65.53	M_234920_296	58.09
1H_82586715	46.37	Bmag518	46.63	3_489536928	45.39	4HL_38170681	57.07	5HL_43862216	77.74	6HL_36155644	66.49	7HL_20380929	58.14
1H_69139092	46.72	Bmac93	46.81	3HL_1279964	46.11	4_261703040	57.23	5HL_18867551	78.52	M_1581573_816	67.40	M_73771_1811	58.25
1H_22955049	47.46	2_147380064	48.41	M_97612_856	46.22	4HL_23617323	58.02	M_1849165_384	78.89	6HL_24721940	68.26	7HL_33935949	58.74
1_167351152	47.87	2_285259057	49.79	3_486640344	46.69	4HL_16624993	58.36	5_167950701	79.36	M_133598_225	68.82	7_528531136	60.07
1H_51202448	48.23	2HL_30717778	50.56	3_486632809	46.87	4_434123085	58.82	M_282476_1183	79.77	M_67547_965	69.10	M_1622733_603	60.31

1H_81216340	48.71	M_128359_1045	51.11	M_158884_25	47.47	4HL_5092076	59.54	6_423872859	80.45	6HL_29033848	69.53	7_519325233	61.05
1_188266189	49.07	2HS_35845316	51.94	3_484679524	47.70	4_430947935	60.56	5_158847739	81.56	6_480444698	70.85	7HL_41147678	63.98
1H_65762157	49.58	M_80184_1375	52.51	3HL_33828484	48.00	Bmag490	62.34	5HS_21385587	82.97	3HL_30556025	72.24	7_521973920	64.59
1_97709789	50.11	2HL_17013042	52.61	3HL_33363575	48.29	Bmac186	62.70	5_208212394	84.28	6_480485322	73.72	7HL_28107982	64.77
1_113838203	50.36	2HS_5593596	53.96	3_482018807	48.92	4_431398963	64.43	5_142876283	85.23	6_480356150	74.15	7HL_8312277	64.95
1H_68973217	50.86	2HL_14077718	55.39	3HL_17280395	49.90	M_1596497_1560	65.03	5_208239424	86.18	6_472829987	74.82	7HL_13143105	65.18
1_113838210	51.84	2HL_33276866	55.78	3HL_28498322	50.43	4_429615338	65.64	M_193887_1076	87.06	M_227287_599	75.21	7HL_37199773	65.75
1H_73559241	52.89	2_362750355	55.97	3HL_15852231	51.48	4HL_44314448	66.16	5HL_23411605	89.25	6_473076290	75.72	7HL_4313756	66.21
1H_58188215	53.86	2_146189440	56.20	3HL_33151823	51.74	M_1606706_765	66.63	5HS_10059836	90.76	2HL_24372145	76.07	7_501748124	66.67
1_113807278	54.36	M_308169_552	56.48	3HL_45910009	52.07	4HL_17692246	67.08	5HS_21724907	92.33	6HL_40780311	76.24	7HL_39503073	67.00
1H_22104337	55.45	M_132750_2128	56.83	3HL_15374972	52.55	4HL_4256734	67.26	5HL_45333334	93.03	6HL_26706644	76.61	M_125216_531	67.43
1_191974362	55.83	2HL_38135646	57.28	3HL_48064911	55.41	4_411663608	67.26	5HS_16248377	93.59	GBM1256	78.13	M_96819_188	67.93
HvM20	56.33	2HL_28612471	57.35	3_474974240	56.31	4_423550415	67.41	5HL_17848298	94.14	6HL_6355520	79.52	7HS_35867270	68.31
1H_67428199	56.61	2HL_40313424	57.71	3HL_34537138	56.99	4_430951797	67.76	4HL_44023327	95.11	6_480507072	79.95	7HL_29967547	68.44
1H_59227174	56.92	2_287559067	57.92	3HL_24669457	57.64	M_325150_1504	68.29	5HS_11608473	95.89	6_468938258	80.46	7HL_3360534	69.18
1_154836162	57.28	M_193322_318	58.11	3_471571393	57.87	4HL_15111896	69.35	5HS_24357909	96.80	M_145223_1780	80.57	M_363857_407	70.04
1H_60459469	57.51	2HS_30524211	58.62	3HL_2943510	58.13	M_300241_491	70.47	5HL_12670406	97.74	4_79532838	80.89	7HL_37641944	70.43
1H_6665309	57.82	2HS_17957603	59.05	Bmag225	59.17	2_508501818	71.23	5_177678359	98.73	M_1625128_432	81.77	7HL_27160293	70.75
1H_66531625	58.47	2HS_14472585	59.15	3_449366069	65.26	4_379247469	71.62	M_290866_2476	99.61	6HL_29286452	82.72	7_499286221	70.75
1_215934943	59.57	2HS_31394907	59.20	M_60564_192	65.59	4HL_20849278	71.62	M_212822_493	100.28	6HL_27550031	83.35	7HL_18681541	71.09
1_141406485	60.40	2_222165120	59.20	3_452489880	65.59	4_383437046	71.74	5HL_22043160	101.67	2HL_44342203	83.95	M_149272_1266	71.65
M_113599_2631	61.40	2_106064968	59.28	3HL_29437708	66.06	Bmac30	72.19	5HS_16446198	102.19	2HS_26945700	84.71	7HL_15512843	71.89
1H_19218707	62.84	2_120687829	59.44	3_453794177	66.48	4_407308714	72.96	M_191556_3625	102.54	6_451975519	85.17	7_500618431	72.18
M_2579923_225	64.13	2HS_6277837	59.61	3_444827923	66.58	4HL_46272554	73.13	5HL_44466712	103.19	M_93290_2008	85.56	7_486000975	72.51
1H_31926957	64.81	2HL_37394745	60.00	3_449368974	67.01	5HL_40573685	73.80	5HS_15859296	103.54	6HL_26059439	85.91	7_486001569	72.71

1_147573318	64.95	2HS_14637826	60.43	3_449419945	67.95	4HL_33997002	74.39	5HL_17760388	103.79	6_442589206	86.03	7HL_38039117	73.01
1_160044163	65.71	M_2564099_1111	60.89	3_445732233	69.05	M_228899_805	74.86	M_109322_807	103.96	6_403040676	91.02	7HL_31396537	73.56
M_126455_423	66.11	2HS_35831478	61.70	3_441949992	69.34	4HL_15475917	75.52	5HL_27068238	104.21	6_413199603	91.43	7_489795725	73.63
1H_82612441	66.19	2HL_30880394	63.04	7HS_39715161	69.68	M_92618_566	76.59	5HS_27679695	104.49	6_415295571	91.61	7HL_25508816	74.02
1H_51201201	66.32	2HL_24337207	63.86	3HL_15283896	70.16	4HL_44079621	77.47	5HL_46081168	104.86	6HL_13457511	91.69	7_485717629	74.60
M_150966_1178	66.93	2HL_49027260	64.59	M_125452_800	70.93	4HL_5909211	77.59	5_200109411	105.36	6_372848417	92.18	M_85228_1401	75.00
1H_45321672	67.81	2HL_43960788	65.65	3_434559804	71.40	M_330520_709	78.06	M_121292_767	105.97	6_408183396	92.52	7_489783222	75.30
M_2563471_507	68.41	2_451183747	66.51	3_434559075	71.89	M_250247_835	78.82	5HS_12487361	107.18	6_411874656	93.20	7_486834197	75.52
1H_44785701	69.03	2_410688104	67.26	M_125452_3570	72.55	M_147172_2403	79.25	5HL_28625859	107.71	M_367945_543	94.16	7HS_40239648	75.67
1H_59785057	69.28	1H_24966320	67.67	Gms116	74.04	6HS_34955464	79.72	5HL_51066705	108.57	6HL_32472005	94.86	7HL_18904683	75.75
1H_63807750	69.69	2_438686337	67.76	M_148546_924	75.68	M_126980_1619	79.91	5HL_32846580	109.00	M_200155_412	95.84	7_500661278	76.14
1_121770457	70.09	2_406934594	68.18	M_2549593_140	75.83	1H_70968226	81.34	5HL_20217360	109.29	6HL_14568415	96.56	7HL_6800849	76.63
1H_75517167	70.57	M_1999039_479	68.47	3_434451394	76.42	4HL_4337750	82.33	5_182351049	109.50	6HL_26939216	97.90	7HL_27748871	77.31
1H_40713213	71.32	2HL_2742232	68.75	3HL_41024310	76.78	4HL_4336601	83.39	5HL_43093388	109.71	6HL_1116639	98.95	7HL_26774972	77.97
1H_13695822	71.57	2_426554428	69.02	3HL_49369809	77.80	4_268295136	85.01	5HS_16459563	109.93	M_2548146_2058	99.26	7HL_18740873	78.07
1_243751360	72.33	2HL_18957514	69.66	3HL_47371940	78.23	4HL_31348524	86.64	M_156886_459	110.22	6HL_25017621	99.48	5HS_28800141	78.63
M_214168_632	72.59	2HL_20952058	70.60	M_78479_1593	78.40	4HL_37729220	86.91	5HS_15933224	110.69	6_485528864	99.66	GMS46	79.71
1_201848085	73.59	2HL_26347197	71.44	3HL_21306461	78.69	4HL_28043578	87.34	5HS_24463022	111.20	1H_87925591	99.86	7_494149861	80.79
1H_61681644	74.55	1H_84464771	71.93	3HL_21303890	79.19	4_311821861	87.55	5_146466415	112.07	6HL_24546503	100.31	7HL_24505608	81.26
1H_46122499	75.05	2HL_19588550	72.11	3HL_39880616	79.73	4HL_40097704	87.70	5HS_19782223	112.77	6_429287001	100.83	7_481164032	82.20
1_129743777	75.25	2_439032332	72.28	3_440999767	80.40	M_146649_4	87.99	5HS_9589875	113.52	M_1661027_233	102.03	M_297541_686	82.60
1H_274731	75.94	2HL_44835824	72.64	3HL_37773053	80.61	4HL_28208411	88.76	M_116376_1416	114.55	6HL_39991404	102.75	7HL_39485022	82.68
1H_74505403	77.76	2HL_10858514	73.14	3_426808044	81.08	6HL_39131112	89.65	M_121125_1375	115.07	6HL_31282585	102.99	7_470854903	82.86
M_85194_2247	78.25	M_223185_321	73.63	3HL_8500939	81.43	4HL_42790942	90.08	5HL_46910494	115.93	M_2086269_498	103.17	7HL_33306869	83.18
1H_61373505	78.40	2HL_31367082	73.85	3_411835196	82.05	4_282929353	90.42	M_182396_1923	116.38	6HL_34025772	103.31	7HL_26774979	83.40

1_186628692	78.68	2_255716516	74.05	3HL_3720522	82.99	M_74213_950	91.06	5_90206977	117.32	6HL_14584491	103.43	M_229649_670	83.93
1H_279094	79.00	2HL_38047576	74.17	3HL_13945592	83.29	4_203374245	91.52	7_158082443	118.30	6HL_13079821	103.51	M_1586526_5	84.02
1_239463847	79.27	2HL_47888627	74.33	3HL_45339617	83.51	4HL_38836656	92.85	5HS_16277353	119.66	6_382186017	103.51	7_467497351	84.10
1H_74505195	79.53	2_370819614	74.64	3HL_27247338	83.60	M_146266_881	94.15	5_237332705	121.17	6_466619694	103.57	7HL_19193116	84.35
1H_60650513	79.78	2HL_25534662	74.74	3HL_11561307	83.60	4HL_13162022	94.53	M_120163_805	122.08	6HL_36077879	103.71	6HL_40682614	84.69
M_131428_1439	80.03	M_207663_343	75.39	3_403275941	83.78	4_366310571	94.61	5HS_4157152	123.53	6HL_13081358	104.14	7HL_6538740	85.09
1H_21840444	80.60	2HL_28152271	76.29	3HL_26328866	84.34	4HL_42047336	95.00	M_213355_436	124.63	6HL_13081351	104.69	7_471932456	85.47
1H_54309325	80.68	2HL_18855931	77.46	3HL_16401098	85.12	4HL_4616362	95.68	5_95944672	125.36	6HL_35014660	105.19	M_69439_721	85.89
1H_70258462	81.26	M_223185_603	78.98	3HL_6379690	85.54	4_312324981	96.26	5HL_29788070	126.05	6HL_35014667	105.98	7HL_30474140	86.47
1H_3132281	81.76	M_167678_1523	79.80	3HL_25561608	85.54	4_260177243	96.79	3HS_810381	126.65	6HL_11679052	107.11	7_469557232	87.11
1H_13600752	82.23	M_256210_824	80.87	3HL_31657066	85.54	4HL_34427461	97.25	5HS_5497569	127.40	6HL_4483655	108.62	7HL_37824340	87.76
M_164333_1071	82.81	2_310280015	81.97	3_396805813	85.85	4_263403559	97.78	3_504149172	128.06	6_405096283	109.34	7_460649517	88.19
1H_65837753	83.68	2_341491653	81.97	3_490323212	85.85	4HL_11548111	98.64	M_1821326_708	128.72	6_385523543	109.52	7HL_7354399	89.32
1H_14055800	84.70	2_386639838	82.39	3_386621954	85.85	4HL_22242513	99.18	M_167216_153	129.47	6_369199540	111.17	7HL_28006539	89.92
1H_63161731	85.83	M_217052_340	83.23	3HL_39017952	85.95	7HL_32686430	99.76	5_243757730	130.24	6HL_31455714	111.74	7_446906010	90.37
1H_51696294	86.27	2_596664618	83.99	3_389354753	86.57	4HL_44654768	100.36	M_119826_513	130.62	6HL_27000128	112.04	7HL_24888341	90.83
1H_65535529	86.43	2HL_25536047	84.50	6HL_25349295	87.77	M_167686_4294	101.74	5HL_38619521	130.82	6HL_7718387	112.38	7HL_29401655	91.38
M_225757_1026	86.58	2HL_44664414	84.94	3HL_19389223	87.99	4HS_24316190	103.04	M_302404_257	131.34	6HL_16162419	113.00	7_428184911	92.10
Bmag211	86.70	2HL_44788406	85.21	3HL_45333061	88.68	4HS_19600191	103.91	5HS_14987153	132.20	M_437770_1111	113.69	7HL_27996637	92.62
1_69741663	86.80	M_207663_1931	85.93	3HL_13755803	89.26	4HS_39712945	104.33	5HL_6073490	133.03	6_392210201	114.63	7HL_39750192	92.90
1H_46868529	86.90	2_394340366	86.58	3HL_5216529	89.60	M_2551499_864	104.80	M_282638_2707	133.62	6_419633203	115.37	7HL_10412411	93.12
1H_2112459	87.02	GBM1218	87.51	3HL_14205585	89.96	M_72645_4588	105.24	5HL_33592928	134.19	6HL_21906841	115.67	7_446872355	93.38
M_1607548_59	87.12	GMS3	87.51	3_217506992	90.33	4HL_4823802	105.40	1H_66366382	134.64	6HL_5968146	116.48	Bmag746	93.97
1H_59768568	87.12	Bmag829	88.11	3_374239627	90.67	4HL_46501110	105.50	M_321019_192	135.66	6_369141566	117.09	7_318484923	94.70
1H_81710076	87.12	2HL_39558214	89.61	3_362874381	90.83	4HL_2479367	105.58	5HL_39150214	136.66	6_371118902	117.45	7HL_17732243	95.33

1H_71315495	87.12	2HL_23335246	90.23	3_267212934	91.07	4HL_45735779	105.68	5HL_49399870	138.06	6HL_24599024	117.69	M_104241_836	95.79
1H_51860442	87.19	5HL_30259839	91.43	3_352516259	91.48	4HL_29929524	105.81	4HS_28052540	138.78	6_354112389	119.62	4HL_39191572	95.89
1_192265578	87.57	2HL_23593797	92.04	M_1580553_1035	92.23	M_304997_83	105.99	1H_81979810	139.72	6HL_28778393	119.97	M_100388_582	96.15
Bmag770	88.97	2HL_26409007	92.84	3HL_42463820	92.91	4_198146445	106.22	M_69912_1020	140.35	6HL_25692192	121.95	7HL_22170668	96.33
1_79061502	89.94	M_1606689_1135	93.24	3HL_4465853	93.38	4_200841878	106.73	5HL_43331862	140.81	6HL_36082630	122.55	7HL_41894552	96.45
1H_3416230	90.55	2HL_24094592	93.37	3_369845685	93.57	4HS_11808880	107.48	M_1634918_588	141.07	6_295566586	123.12	7_424838106	96.51
1_86437113	91.15	2HL_42741686	93.37	3HL_6015573	93.76	3HS_22987940	107.69	5_211041692	141.48	6HS_905513	123.96	7_440111505	96.92
1H_63110764	92.07	M_294977_1390	93.42	3HL_15822689	93.94	4HL_34930707	108.06	M_65287_3266	142.03	6HS_14436965	124.10	7HL_39790713	97.39
1_414284041	93.06	2HL_2741060	93.71	3HL_2694409	94.11	4HL_45649291	108.71	5HL_36921809	142.81	6HL_23850671	124.82	1H_54211777	97.85
1_70081144	94.44	M_230635_804	94.41	3HL_19606269	94.36	M_140524_2639	109.34	5HL_3649575	143.56	6HL_30097652	125.47	7HL_39790706	97.98
1_69995154	95.23	M_54203_5996	95.12	3HL_32665027	94.60	4HL_35946015	109.71	5HL_35363259	144.08	6HS_11428410	125.73	7_431414178	98.14
M_248244_1456	95.86	2HL_27432067	95.84	1_446245465	94.81	M_180821_933	110.45	5HL_29047107	144.48	6_360051240	125.86	7HL_33672358	98.60
1H_76945612	96.25	2HL_43859802	96.02	3_381989792	95.36	Ebmac906	110.77	M_70688_2113	145.30	6_254867927	126.37	7_431349278	99.20
1H_63044931	97.09	2_447773331	96.41	3_379024546	95.79	4_166903149	110.89	M_322077_429496	145.85	6HL_26708031	127.04	7_256493146	100.89
1H_47641355	97.71	2HL_37542979	97.81	3HL_7660023	96.39	3HS_12473800	111.01	M_146187_940	146.68	6HL_1993657	127.58	M_168364_499	101.44
1_74125493	98.42	2_521014801	99.14	3HL_16015449	97.53	4HS_39092168	111.27	M_1634918_581	147.41	6_307582762	128.07	7HS_42314946	101.77
1_71832171	98.69	M_173221_716	99.95	3_219109045	98.35	4_231149779	111.94	M_302796_98	149.08	6HL_23405967	128.71	7HL_11953678	102.47
1H_50989596	99.02	2HL_16566180	100.34	3_428733082	98.60	4HS_27244737	112.65	5_301063594	149.84	6HS_18700041	129.66	7_276974619	103.23
1_64786750	99.41	2HL_23870380	101.25	3_371192089	98.72	4HS_22306623	112.83	5_309377165	150.60	6HL_20672652	130.65	7HS_9727363	103.35
Bmag718	99.78	2HL_134136	101.79	M_227450_667	98.86	4_163898518	113.23	5_333691837	151.43	6HL_20105418	131.15	7HS_38161179	103.92
1H_71490536	100.21	2HL_18527860	103.41	3_219205628	99.78	4HS_35038521	113.68	5HL_10821972	152.62	6_136865314	131.34	M_172321_1859	104.33
1H_48137486	100.84	2_473837037	104.36	3HL_43341146	100.55	4_85978384	114.76	5HL_36003694	153.69	6HS_15595303	132.50	7HL_32111786	104.45
1H_36913991	101.72	6_149903294	107.32	3HL_19802894	101.46	4_172632686	115.02	5HL_42421219	154.22	1_461094537	133.29	M_89435_978	104.79
1_63836089	102.40	2_479999250	108.04	M_128042_720	102.11	M_242005_79	115.43	5HL_13495684	154.62	M_413056_642	133.67	M_298899_323	105.44
1H_52636104	104.06	2HL_42857061	108.69	3HL_13120701	102.83	4HS_28702663	115.65	M_81421_1318	155.35	6HS_10840212	133.81	7HS_15895352	106.07

1H_11727006	104.85	2_386606309	109.17	M_115827_1345	103.31	4HL_29931449	116.03	5HL_19633581	156.16	6_167517556	133.92	7_266425095	106.69
1H_28288036	105.67	2HL_32765002	109.57	3_181578804	103.68	4HS_4815132	116.23	5_306133226	156.86	6_119236872	134.03	1H_82374297	107.52
1_88134541	107.02	2_411931129	109.97	3HS_24352223	104.01	4HS_19718929	116.78	M_198558_704	157.68	6HS_4428068	134.35	7HS_31863896	108.08
1H_46993132	107.58	2_481879811	111.39	3HL_30928831	104.33	4HS_37856342	117.64	4HL_36557878	158.37	6HS_21293212	134.73	7HS_25452946	108.49
1H_35430201	108.03	2_488526484	111.54	3HL_5729862	104.73	4_235768001	118.08	5_333690029	158.67	6_167519527	135.26	7HS_35580690	109.26
1_126870806	108.40	M_118538_318	111.66	M_328622_766	105.26	M_75617_4898	118.55	5HL_18262105	159.33	6_119237390	135.85	M_310867_507	110.15
1H_68770353	108.67	2_481833725	112.17	M_1604359_2677	105.33	M_1586023_526	119.28	6_430872418	159.87	6_100251183	137.40	7HL_36999769	110.49
1H_52481961	109.24	2_485077422	112.68	3HS_1235507	105.94	7HL_15514967	120.18	5HL_32148262	160.09	M_1588185_708	137.61	7_270366894	110.82
1H_21410475	109.43	2HL_10917142	113.05	3_115656395	106.38	4HS_32466333	121.56	5HL_22883152	160.38	6HS_29527846	138.12	7HS_26745260	111.40
1H_21409814	109.80	2HL_16222169	114.15	M_131037_1732	106.59	4_185392211	123.54	5HL_4248479	160.92	6HS_1861379	139.59	7HS_26362969	111.97
M_299738_727	110.37	2HL_13832944	115.84	3HS_36393149	107.17	M_168917_926	124.99	M_1645985_1227	161.72	6HS_21863145	140.83	M_171247_237	112.57
1H_32618947	112.03	2_480244865	116.28	3_199957740	107.83	4HS_21093462	125.25	Bmac303	162.76	6HS_19809554	141.83	7HL_36983527	113.20
1H_34522869	113.88	2_519929645	116.83	3HL_18997117	108.69	4HL_47348553	126.12	Bmag323	162.76	M_429316_458	142.24	7_286729861	114.09
1H_34524346	114.50	2HL_13831365	117.41	3HS_1851613	109.29	4HS_21348659	126.93	M_96665_103	163.63	M_75484_481	142.72	7HL_31175469	115.22
1_58149966	115.68	2_456666117	118.78	3_117826936	109.91	4_121425641	127.16	5HL_49625566	164.84	6_122597999	143.01	5_496886371	115.85
M_192224_1318	116.36	2_497016140	119.06	Bmag138	110.51	M_142742_467	127.35	5HL_33488756	165.56	6HS_23800167	143.11	M_254133_378	116.44
1_41186142	117.01	2_506545106	120.49	Bmac129	111.35	M_56490_2564	127.54	5HL_42668326	166.28	6_153928349	143.66	7_302813681	117.06
1_41342487	117.43	2_496663791	121.59	3_252652374	111.92	M_80588_361	127.77	3HS_38438843	167.04	6_142602565	143.96	7HS_21525102	117.64
1H_84570760	119.19	2_514353957	122.98	3_222316683	112.65	4_98701946	128.40	M_224427_816	168.07	6_36795707	144.55	7HS_31095616	118.03
1_38969627	120.10	2HL_13648618	123.44	3HS_26700700	113.38	5HS_25881971	129.34	5HL_31814320	168.65	6HS_14342863	145.11	7_262149691	118.25
1_41975622	120.91	2HL_13649329	123.73	3HL_4462181	113.47	4HS_17482435	129.83	5HL_44103275	168.90	6_148316429	145.64	7_234342476	118.32
1_30871398	125.97	2_521439621	124.30	3HL_36837491	114.43	Ebmag781	130.58	5_342998304	169.13	7HL_32848991	146.41	7HS_39680044	118.80
1H_74333028	126.63	2HL_7395625	124.44	M_121700_1366	115.34	4HS_6004759	131.17	5_370389808	169.55	6HS_13065896	146.99	7_268311781	119.01
1_12786191	127.55	2HL_18970523	124.60	2HS_36129225	116.01	4HS_18042559	131.71	5HL_35148946	170.23	6HS_32749126	148.38	7HS_29124063	119.36
1_19877648	128.19	2HL_22930294	125.19	6HS_30881605	116.94	4_93134040	133.02	5HL_23372706	171.03	GMS6	150.15	7HS_423705	119.41

1H_40868061	128.38	2_527241334	126.00	3HS_13376040	117.58	4_244618842	133.77	5HL_36403860	172.37	3HL_21642944	150.69	7HL_22161891	120.40
1H_75978121	128.74	2HL_22930005	126.97	3_106355000	118.02	4_48614035	135.18	1H_59344714	173.76	M_229673_418	151.02	7HS_6744377	121.40
1_21963834	130.83	2HL_17075593	127.35	M_56999_3219	118.23	M_1574682_242	135.73	M_1577838_3038	174.20	6HS_14944477	152.41	7HS_21726812	122.12
1H_55583778	132.59	2_522610509	127.96	3_122822375	118.92	4_48624357	136.43	Bmag337	175.56	6HS_27140927	152.87	M_325776_4784	122.52
1_19825452	133.38	2HL_34260490	128.68	3HS_7624882	119.74	4_45907643	137.78	HvLEu	177.30	6HS_14897087	152.98	M_104409_1533	122.82
M_1574335_133	139.12	2_524782265	129.63	3HS_26938494	120.28	M_232034_80	138.68	M_188794_1190	178.50	1H_70160136	153.07	7HL_33754146	122.97
1_11525078	140.83	2HL_41874761	131.10	M_1573468_3132	120.71	4HS_14418535	139.16	5HL_3126208	178.91	6HS_27453116	153.62	7HS_13114449	123.12
1H_30210492	141.25	2_531230596	131.46	M_123041_952	120.85	4HS_16506867	139.44	5_221233297	179.35	M_1619409_276	153.98	7HL_32655325	123.24
1H_11559496	141.53	2HL_43143355	132.50	3HS_25731240	121.43	4_42378533	139.62	M_320650_521	179.88	6HS_13955828	154.31	7_394187342	123.33
1_9928110	141.81	2_534686550	132.96	4_45462104	121.91	4_26092518	142.50	5HL_23991301	180.52	6_61157407	154.53	7_325705605	123.37
1_2849437	143.17	M_149956_1482	133.61	3HS_35126372	122.47	4_28741355	143.67	M_320650_528	180.90	6HS_12971159	154.75	7_394184659	123.37
1H_2745781	145.04	M_165611_94	136.79	3HS_14955052	123.22	4HS_32009949	145.47	5_429870353	183.82	6_61144426	154.93	7_202879929	123.37
;ENDOFGROUP		2_535589467	137.42	3HS_25915649	124.17	4_19543214	146.25	5HL_37078543	185.37	6HS_26273874	155.11	7_311871132	123.44
		1H_83810983	137.73	3HS_35585265	124.54	3HL_12431542	146.39	5HL_8494280	185.79	1H_83380277	155.77	7_380865149	123.56
		2_540559791	138.18	7HL_39841684	125.10	M_153521_1223	146.50	5HL_11658154	186.14	6HS_25779094	156.13	M_129326_472	123.77
		2HL_766321	138.78	3_142888281	125.58	M_1605646_794	146.57	5HL_33815718	186.64	6HS_16621012	156.26	7HS_32890650	124.12
		2_544135082	139.15	M_224438_795	126.79	4HS_38761945	146.67	5_412651332	187.53	4_399455275	156.36	7HS_20285665	124.45
		2HL_23978934	139.58	3HS_8199020	127.31	4_16553551	147.72	5_416204256	188.80	6_49398767	158.65	7HL_24981369	124.83
		2HL_37245721	140.32	3HS_35241834	127.45	4HS_28564347	148.36	5_417419714	188.96	6_51238374	159.56	7_394208044	125.10
		2HL_31619458	141.75	3HS_38268253	127.59	M_2553137_482	151.34	5_415005971	189.34	6_53661541	159.78	1H_81411360	125.82
		2HL_23067013	142.99	3HS_34810522	127.70	4_16819133	151.97	M_148497_192	189.73	Bmag103	160.21	7HS_26227899	126.49
		2_561951308	143.88	3HS_19353169	127.89	4HS_9277108	156.16	5_428226858	194.01	GBM1212	161.04	7_348795436	127.19
		2HL_44347520	144.58	3HS_30849038	128.43	HvM40	156.31	5HL_1829158	194.86	6_33577280	161.92	7HS_15303153	127.72
		2HL_20046952	145.57	3_168355506	129.32	4_12041406	156.45	5_424540233	196.96	6_47097505	162.71	7_291301444	128.35
		2_558613423	147.31	3HS_25993959	129.57	4HS_38146635	156.57	5HL_12771179	199.63	6_44153505	163.07	7HS_27437325	129.02

		2_576439205	157.20	3_168380765	130.02	M_120736_1101	156.69	5_438033432	201.50	M_63492_429	163.59	7HS_31297929	129.52
		1H_58333588	158.79	3_164913353	130.83	4HS_21913031	157.59	5HL_6105296	202.20	6HS_25127741	168.20	7HS_25905506	130.00
		2HL_28631837	159.76	3_151654877	131.25	4_8019773	158.06	5_226253827	202.87	M_139325_912	168.64	7HS_35889676	130.18
		2HL_34903597	160.76	M_166308_3474	131.99	4_8073993	158.50	5_436205970	203.25	6_29134076	169.32	7HS_30003071	130.93
		M_537170_780	161.45	3HS_32884333	132.41	M_1611184_441	161.19	5_435781539	203.83	6HS_6762219	169.59	7HS_21578173	131.12
		2HL_18227895	161.99	3HS_31172493	133.10	M_1635498_753	161.91	5HL_25802367	207.85	M_255849_392	170.35	M_152709_1608	131.32
		2_579576154	162.44	3HL_9800712	133.41	M_282112_578	162.79	5_443547683	208.36	6_25200756	171.27	7_265172609	131.66
		2_568727199	163.27	3_342720039	133.74	4_3860802	164.17	5HL_37477531	208.95	GBM1215	172.36	7HS_22488622	131.97
		2HL_13810728	164.31	4HL_23236962	134.36			5_442118440	209.47	6_24969652	172.75	M_107505_2072	132.59
		2_578947595	165.30	3HS_15347221	134.78			5_450805227	209.95	M_279413_743	173.11	7HS_17906516	133.19
		2_585157702	166.22	4HL_51597838	135.47			5_441132308	210.45	3HL_48888709	173.41	M_101938_837	134.41
		M_1020_157	166.97	M_114292_1468	136.07			M_200683_71	212.15	6HS_23958993	176.36	7HS_33828406	135.00
		Ebmag793	170.04	M_169425_661	137.09			M_133537_1411	213.81	2_625859552	176.91	7HS_12989483	135.05
		GBM1149	170.87	3HL_28233421	137.83			5_455691597	214.70	6HS_34488883	178.47	7_382544975	135.36
		2_588819729	175.09	3_202935520	137.97			2_91618222	215.29	6_14536026	179.49	7HS_29196961	136.21
		2_588885691	175.90	3HL_9802508	138.53			5_460258834	216.96	Bmag500	179.68	M_126443_1107	137.28
		2HL_14160939	176.99	3HL_30415730	138.95			5_460285749	217.82	M_195029_1219	180.91	7HS_8348321	138.20
		2_588885698	177.68	3HL_12333356	139.37			5HL_15003501	218.19	M_95671_1878	183.20	1H_61339959	138.27
		2_598440313	183.47	M_1804790_860	140.03			5_464856478	218.60	6HS_10274897	183.70	4HL_50830351	138.91
		2HL_19715945	183.83	3HL_6125197	140.09			M_1573849_850	218.93	3HS_30515537	184.32	7_319506952	139.84
		2_598509820	184.06	M_149429_1285	140.68			5_457074676	219.20	6HS_7381030	185.43	7HL_28498244	140.52
		2_598551866	184.64	3_117331152	141.08			5_472488768	223.12	6HS_23929119	188.54	7_332338124	141.21
		2_594496551	184.94	3_212034112	141.51			5_475126791	223.69	6HS_10046074	189.38	7HL_18225538	141.35
		2_598635476	185.41	M_106257_1414	142.25					6HS_15502602	189.64	7_266426884	141.78
		2_599214360	186.97	3HL_2971556	142.72					6HS_5930053	195.77	7HL_38122468	141.93

		2_598741132	187.57	3_228358690	143.15					M_285479_975	196.56	7_363854703	142.10
		2_600701693	188.30	3HS_32545487	143.51							M_249458_259	142.26
				3_260593928	144.54							7HS_4123979	142.42
				4HL_14912109	145.33							7HS_11473727	142.53
				3_195437549	146.06							7HL_32593133	142.60
				3_85361523	146.60							M_378389_1034	142.65
				3HL_17901650	147.31							7HL_34691420	142.65
				3_115656388	148.06							M_197346_1107	142.65
				3HL_39867477	148.61							7_242293096	142.65
				3HL_46409485	149.04							M_95954_1839	142.65
				3_252656251	149.61							7HL_19514027	142.69
				3HL_4528686	150.19							7HS_23764153	142.74
				M_124614_1992	150.53							7_231161159	142.82
				3HL_36459271	150.92							7HL_19464259	142.92
				3HL_17169545	151.05							7HL_19514020	143.01
				M_170332_914	151.42							7_379313804	143.11
				3_117329141	152.07							7HL_39611722	143.24
				3HS_1849547	152.62							7HS_28646469	143.39
				3HL_6035493	152.94							7_275021515	143.76
				3HS_26388351	153.15							7_253830472	144.07
				3HS_35720654	153.30							7HL_41207635	144.41
				M_68399_2570	153.40							7HS_18303337	145.06
				3_232531541	153.55							7HS_38071125	146.07
				3HS_29284311	153.76							5HL_40328673	146.86
				3HS_21692681	153.91							7_197072870	147.60

				3_260516488	154.02							7HL_1406395	148.00
				3HL_22314417	154.08							M_164402_210	148.40
				3HL_45008247	154.15							7HL_36320136	149.07
				3HL_33458679	154.24							M_96111_1679	149.42
				3HS_14684481	154.37							7HL_6335336	150.11
				3HS_10098927	154.53							Bmac31	151.09
				3HL_22346532	154.73							7_249275418	151.48
				3HS_36183965	155.01							Bmac167	151.48
				3_230154353	155.32							Bmag217	151.48
				3HS_33787164	155.52							2_287569753	151.92
				3HS_20423735	155.92							7HL_29535430	152.24
				3_351352181	156.25							M_90598_706	152.84
				3HL_49792701	156.48							6HL_26291109	153.44
				3HS_8311834	156.62							M_1645585_244	154.04
				3_175635979	156.71							7HS_25585637	154.94
				M_286283_706	156.77							7_169171765	155.79
				3HL_26275170	156.83							M_92577_622	156.15
				3HL_38044646	156.89							2HS_16886086	156.50
				3_564584365	156.94							M_231959_191	157.12
				M_189878_37	156.94							7HS_30953596	158.65
				3HS_21357171	156.94							M_225539_34	159.11
				3HL_35306249	156.99							7_227758562	159.88
				3HS_1055659	157.07							7_276875544	160.01
				M_363498_76	157.14							M_247532_1263	160.15
				3_327391418	157.23							7HS_29625706	160.40

				3HS_22945153	157.34							7HS_33151126	160.97
				M_191167_715	157.46							M_262022_735	163.92
				6_299289413	157.57							7_171033057	164.63
				3HS_19418212	157.67							7_194302352	165.03
				3HS_9525966	157.79							7HS_33962273	165.18
				3HL_8293691	157.97							7_194262618	165.25
				3_166537144	158.18							7HS_14000381	165.59
				1H_52413474	158.38							7HS_5799924	165.67
				M_1876186_1745	158.56							Bmag571	166.29
				3HL_38461305	158.74							Bmag900	166.77
				3HS_20733519	159.39							7HS_17452871	167.28
				3_226759716	160.04							M_11043_65	167.62
				3_311020965	161.17							7_146904388	167.94
				3HS_35296545	162.33							7_144943549	168.30
				3HL_14365348	162.65							7HS_5988002	168.48
				3_300676410	162.99							7HS_20967504	168.77
				M_1793953_954	163.61							7HS_38089752	169.59
				3HL_10773026	164.16							7_131382045	171.15
				3_237895833	164.91							M_2556064_1255	172.26
				M_280918_1333	165.42							7_152087825	172.73
				M_460647_732	166.08							7_180156097	172.81
				3HS_29011616	166.97							7HS_42524160	173.05
				M_148075_2494	167.32							7_180157107	173.46
				3HS_34434545	167.71							7_228780727	174.03
				3_334498383	168.00							7_164701417	174.49

				M_201196_1098	168.67							7_144173681	174.98
				3_201938731	169.50							7HS_24288175	175.26
				3_275423042	169.89							7HS_16914589	175.79
				3_195703173	170.22							7HS_24124059	177.72
				3HL_31281289	170.94							7_175785765	177.87
				3HL_34232600	171.44							7_115993906	178.52
				3HL_14019093	171.79							M_221260_990	179.12
				3HL_46099559	172.22							7_135299038	179.43
				3_205578288	172.68							7HS_24633706	179.63
				3HL_38526556	173.31							7HS_16458224	180.01
				3HL_45008254	174.27							7HS_39727659	180.69
				3HL_25942927	175.76							7HS_7499702	181.04
				3HL_47239478	177.30							7_100916612	181.14
				3_254103649	178.55							7HS_23607469	181.34
				M_2562830_280	179.17							7HS_33683527	183.45
				3_421573021	179.90							7_247969253	183.45
				3HL_40793534	180.29							7HS_21827941	183.75
				3HL_10764412	180.43							7HS_30257307	184.08
				3HL_41163989	180.55							7_87591581	184.55
				3HL_8294472	180.90							7_74542996	184.93
				3HS_22869183	181.26							7HS_10887541	184.97
				3HL_9800668	181.65							7_81565191	184.97
				3HL_18994954	182.22							7HS_15526962	185.10
				3_426707684	183.63							7_68207046	185.27
				M_171790_606	184.99							3HS_37315153	185.43

				4_261711184	185.59							M_128508_616	185.70
				3_168429388	186.33							7_74542989	186.23
				M_174364_930	186.59							7_92359522	186.64
				Bmag6	187.79							7_77944466	187.22
				Bmac67	188.40							7_75744987	190.07
				M_168364_1478	190.30							7_68192567	191.39
				3HS_28871858	191.42							7_66854471	192.72
				3HS_23529315	192.01							7_71636075	193.32
				M_157760_958	192.97							7HS_25923987	194.50
				3HS_5478941	193.44							7_57335292	196.01
				3_74018396	193.82							7_57335299	196.23
				3HS_3937648	202.64							7_54057035	197.84
				3HS_7709043	202.95							Bmag914	198.99
				3_34191994	204.49							7_52685324	199.11
				3HS_1093997	205.74							7_52555807	199.56
				3_28890185	207.01							7HS_2758449	200.45
				2HL_24653676	208.25							7_46081414	201.19
				3_28198240	209.20							5HL_38629580	201.34
				M_112120_1515	211.32							7_41328131	204.52
				3_28802747	211.43							4HL_41496393	205.01
				3_26035945	211.60							2_406786591	205.52
				3_28844640	212.04							7_28180247	208.12
				3_19138070	215.17							7_31360519	208.78
				3_18321105	215.90							7_28180957	209.43
				3_12801553	217.97							7_30270714	210.11

				3_10879419	219.05							7HS_22579543	210.32
				3HS_23386841	219.59							7_24915439	212.18
				3_13048803	220.35							7_24487536	212.80
				3_9347910	222.03							7_24486635	213.15
				3HS_5193202	222.47							7_15215651	216.99
				3HS_5195766	222.91							7_18054889	217.33
				3_8289337	224.21							7_16284976	217.52
				3_8275167	224.73							7HS_25179223	217.82
				3_6555065	226.08							7_17975812	218.12
				3_3120137	226.76							M_188044_1595	218.30
				3_5294954	228.53							M_137201_2559	218.43
				M_92173_5387	229.65							7_16284969	218.51
				GBM1280	230.90							7_13793206	218.87
												7_5696029	219.10
												7HS_41263643	219.35
												Bmag206	219.65
												7_11478211	222.36
												7_11445098	223.03
												7_5257716	224.09
												7_2869142	224.41
												M_94859_1021	224.67
												7HS_22537347	225.53
												7_5539511	226.07
												7_2135815	227.14
